# Genetic variability of five *ADRB2* polymorphisms among Mexican Amerindian ethnicities and the Mestizo population

**DOI:** 10.1371/journal.pone.0225030

**Published:** 2019-12-02

**Authors:** María Guadalupe Salas-Martínez, Yolanda Saldaña-Alvarez, Emilio J. Cordova, Diana Karen Mendiola-Soto, Miguel A. Cid-Soto, Angélica Luckie-Duque, Hermenegildo Vicenteño-Ayala, Francisco Barajas-Olmos, Cecilia Contreras-Cubas, Humberto García-Ortiz, Juan L. Jiménez-Ruíz, Federico Centeno-Cruz, Angélica Martínez-Hernández, Elvia C. Mendoza-Caamal, Elaheh Mirzaeicheshmeh, Lorena Orozco

**Affiliations:** 1 Immunogenomics and Metabolic Diseases Laboratory, Instituto Nacional de Medicina Genómica, Secretaría de Salud, Mexico City, Mexico; 2 Genomic Sciences Program, Universidad Autónoma de la Ciudad de México, Mexico City, Mexico; 3 Hospital Regional 1° de Octubre, ISSSTE, Mexico City, Mexico; 4 Hospital Regional Adolfo López Mateos, ISSSTE, Mexico City, Mexico; 5 Clinical Area, Instituto Nacional de Medicina Genómica, Secretaría de Salud, Mexico City, Mexico; University of Iceland, ICELAND

## Abstract

The Mexican population is characterized by high and particular admixture, and the picture of variants associated with disease remains unclear. Here we investigated the distribution of single nucleotide polymorphisms (SNPs) in the Mexican population. We focused on two non-synonymous and three synonymous SNPs in the beta-2 adrenergic receptor gene (*ADRB2*), which plays key roles in energy balance regulation. These SNPs were genotyped in 2,011 Mexican Amerindians (MAs) belonging to 62 ethnic groups and in 1,980 geographically matched Mexican Mestizos (MEZs). The frequency distribution of all five *ADRB2* variants significantly differed between MAs, MEZs, and other continental populations (CPs) from the 1000 Genomes database. Allele frequencies of the three synonymous SNPs rs1042717A, rs1042718A, and rs1042719C were significantly higher in Mexican individuals, particularly among MAs, compared to in the other analyzed populations (*P*<0.05). The non-synonymous *ADRB2* Glu27 allele (rs1042714G), which is associated with several common conditions, showed the lowest frequency in MAs (0.03) compared to other populations worldwide. Among MEZs, this allele showed a frequency of 0.15, intermediate between that in MAs and in Iberians (0.43). Moreover, Glu27 was the only SNP exhibiting a geographic gradient within the MEZ population (from 0.22 to 0.11), reflecting admixed mestizo ancestry across the country. Population differentiation analysis demonstrated that Glu27 had the highest *F*_ST_ value in MAs compared with Europeans (CEU) (0.71), and the lowest between MAs and Japanese (JPT) (0.01), even lower than that observed between MAs and MEZs (0.08). This analysis demonstrated the genetic diversity among Amerindian ethnicities, with the most extreme *F*_ST_ value (0.34) found between the Nahuatls from Morelos and the Seris. This is the first study of *ADRB2* genetic variants among MA ethnicities. Our findings add to our understanding of the genetic contribution to variability in disease susceptibility in admixed populations.

## Introduction

The Mexican Mestizo (MEZ) population is one of the most genetically diverse populations worldwide due to the admixture between Native American, European, and African populations [1]. In addition to MEZs, the Mexican population also includes a great diversity of Mexican Amerindians (MAs), who were the original settlers of Mexico. The MA people currently constitute 14.9% of the population (15 million), distributed into 68 ethnic groups throughout the Mexican territory [2,3]. Genomic diversity studies reveal vast genetic differences between the MEZ population and most of the continental populations (CPs), as well as between MAs and MEZs [3–5]. Therefore, the Mexican population is characterized by a high and particular admixture.

Recent studies suggest that ethnic diversity may introduce genetic variations that can potentially generate inter-individual differences in disease susceptibility and therapeutic efficacy [6–8]. These findings could be explained within an evolutionary framework, in which the frequencies of specific alleles reflect ancient genetic adaptations that have shifted due to environment and lifestyle differences among human populations [9]. However, most research in this field has been performed among Caucasians [7,10].

The protein encoded by the beta-2 adrenergic receptor gene (*ADRB2*) plays a key role in energy balance regulation and is a target for many drugs that are commonly used to treat different conditions [11,12]. *ADRB2* is an intron–less gene located on chromosome 5q31-32, which is of particular interest due to its impact on the genetic risk for several common illnesses, including obesity, asthma, and cardiovascular disease [13–15]. Notably, *ADRB2* shows great inter-population variability in allele frequencies [16,17]. Since *ADRB2* may have been subjected to balancing selection during human evolution, it is a particularly interesting candidate for evaluating how the genetic structure of a population affects the inter–individual differences in susceptibility to chronic degenerative diseases and response to therapeutic drugs [17,18].

Among the single nucleotide polymorphisms (SNPs) found in the *ADRB2* coding region, the two most studied are the non-synonymous SNPs rs1042713 and rs1042714, which result in amino acid changes at protein positions 16 (Gly16Arg) and 27 (Gln27Glu), respectively. The variant alleles of these SNPs modify the receptor activity at several levels, and may also affect the response to therapies with beta-2 adrenergic receptor (b2-AR) agonist through a mechanism involving agonist-promoted down-regulation of receptor expression [10,19]. Recent reports demonstrate that other synonymous SNPs in this gene can affect RNA stability and thus alter the amount of protein [20]. Accordingly, the variants rs1042717 (Leu84Leu), rs1042718 (Arg175Arg), and rs1042719 (Gly351Gly) have been associated with malaria susceptibility, hypertension, longevity, and asthma [20–23].

Although *ADRB2* gene variants play an important role in disease susceptibility and drug responses, they have been scarcely studied among MEZs [24,25] and there are no previous reports of the geographic distribution of these variants in the MA population. In the present study, we aimed to investigate the distribution of five coding SNPs in *ADRB2* within the MA population, as well as their contribution to the ethnic structure of the MEZ population.

## Methods

### Study population

This study included 2,011 unrelated MAs, belonging to 62 different ethnic groups distributed throughout the Mexican territory, from the Metabolic Analysis in an Indigenous Sample (MAIS) cohort study [3]. The participants identified themselves as indigenous, spoke the same native language as their parents and grandparents, and were born in the same region as their parents and grandparents. Our study also included 1,980 unrelated MEZ adults whose parents and grandparents were born in Mexico. This study was conducted in accordance with the Declaration of Helsinki, and was approved by the ethics and human research committees of the National Institute of Genomic Medicine in Mexico City, Mexico. All participants provided written informed consent, and their confidentiality was preserved at all times.

Since admixture of the MEZ population has generated great genetic diversity throughout the Mexican territory, we also investigated the Amerindian influence on the regional admixture of the MEZs based on the frequency of *ADRB2* polymorphisms. For this analysis, we compared the genotypic and allelic frequencies of the five studied SNPs between 1,851 Amerindian individuals (representing 31 Amerindian groups, each including at least 10 individuals) and 1,980 MEZ individuals matched by geographic region. Both MAs and MEZs were sorted into five geographic regions: North, Central East, Central West, South, and South East [3,4].

### Genotyping

Genomic DNA was extracted from whole blood using the QIAmp DNA Blood Maxi kit (Qiagen Systems, Inc., Valencia CA), following the manufacturer’s protocol. All subjects were genotyped for five SNPs localized within the coding region of *ADRB2*: the non–synonymous SNPs rs1042713 (G/A, Gly16Arg) and rs1042714 (C/G, Gln27Glu), and the synonymous SNPs rs1042717 (G/A, Leu84Leu), rs1042718 (C/A, Arg175Arg), and rs1042719 (G/C, Gly351Gly). Genotyping was performed using the TaqMan Allelic Discrimination assay on an ABI PRISM 7900 thermocycler (Applied Biosystems, Foster City, CA, USA). The genotyping call rate was over 96% in all tested SNPs, and no discordant genotypes were found in samples run in duplicate (15%). The TaqMan results were validated by direct sequencing of random samples from each genotype (10%) using an automated ABI PRISM 310 Genetic Analyzer (Applied Biosystems Foster City, CA, USA) with 100% reproducibility. The MA population cohort had an average Amerindian ancestry of 95 ± 5%, as previously described [3].

### Statistical analysis

Allele frequency comparisons were performed by using a chi-square test with the PLINK v1.07 program [26]. A *P* value of <0.05 after Bonferroni correction was considered significant. To measure the level of population differentiation, individual allelic and genotypic data were used to calculate the Wright’s fixation index (*F*_ST_) using GENEPOP software version 1.2 [27]. Linkage disequilibrium (LD) and haplotype structure were analyzed using Haploview software version 4.2 (http://www.broad.mit.edu/mpg/). All maps were constructed with QGIS software version 2.14, and were modified from the National Commission of Knowledge and Use of Biodiversity (CONABIO) [28]. For the MA population, we estimated the correlation coefficient between the allele frequencies of each variant and various geographic coordinates of the ethnic groups (including altitude, latitude, and longitude), and the significance was evaluated by the Pearson’s test, using R version 3.4.4 statistical software [29].

## Results

### Distribution of *ADRB2* polymorphisms and haplotype analysis in MA and MEZ populations

The allele and genotype distributions of the five presently analyzed *ADRB2* SNPs were in Hardy–Weinberg equilibrium among both MAs and MEZs. We further found that the allelic and genotypic frequencies of rs1042713A (Arg16) were similar between these two populations (*P* > 0.05). In contrast, the frequency of the Glu27 (G) allele of rs1042714 was significantly lower in MAs than in MEZs (*P* = 1×10^−**8**^), and the GG homozygous genotype was not observed in any Amerindian ethnic group. On the other hand, the allelic and genotypic frequencies of rs1042717A, rs1042718A, and rs1042719C were significantly higher in MAs than MEZs (*P* < 0.001; Tables 1 and S1).

**Table 1:** Comparison of allele frequencies of *ADRB2* SNPs in MAs, MEZs and other continental population.

	Allele Frequencies							
SNP (Allele)	Present study		1000 genomes (phase 3)					
	MAs (n = 2011)	MEZs (n = 1980)	MXL (n = 64)	CEU (n = 99)	IBS (n = 107)	YRI (n = 108)	JPT (n = 104)	CHB (n = 103)
rs1042713 (A)	0.47	0.44	0.48	0.35^b^^,^^c^	0.38^a^	0.53^c^	0.44	0.55^a^^,^^c^
rs1042714 (G)	0.03	0.15^b^	0.14^b^	0.47^b^^,^^d^	0.43^b^^,^^d^	0.12^b^	0.06^a^^,^^d^	0.11^b^
rs1042717 (A)	0.51	0.43^b^	0.38^a^	0.19^b^^,^^d^	0.19^b^^,^^d^	0.35^b^^,^^c^	0.46	0.34^b^^,^^c^
rs1042718 (A)	0.50	0.42^b^	0.38^a^	0.17^b^^,^^d^	0.15^b^^,^^d^	0.34^b^^,^^c^	0.47	0.35^b^^,^^c^
rs1042719 (C)	0.53	0.47^b^	0.49	0.26^b^^,^^d^	0.28^b^^,^^d^	0.35^b^^,^^d^	0.54	0.44^a^

a MAs vs other populations *P* < 0.05.

b MAs vs other populations *P* < 0.0001 (Range,10^−4^ to 10^−8^).

c MEZ vs other populations *P* < 0.05.

d MEZ vs other populations *P* < 0.0001 (Range,10^−4^ to 10^−8^).

To obtain a global perspective regarding the behavior of these variants, we compared the presently observed allele frequencies with those reported for CPs in the 1000 Genomes database. Compared populations included Utah Residents (CEPH) with Northern and Western European Ancestry (CEU); Yoruba in Ibadan, Nigeria (YRI); Han Chinese in Beijing, China (CHB); Japanese in Tokyo, Japan (JPT); Mexican Ancestry from Los Angeles USA (MXL); and Iberian Population in Spain (IBS). This last population was included because the European contribution to Mexican genetic admixture is mainly from Spain [5,30].

These comparisons indicated that the frequency of the rs1042713A (Arg16) allele among MAs was similar to the rates reported for MXL, YRI, and JPT (*P* > 0.05), but significantly different from CEU, IBS, and CHB (*P* < 0.05). Remarkably, MAs exhibited the lowest frequency of the rs1042714G (Glu27) allele (0.03) compared to all CPs [MXL, 0.14; CEU, 0.47; IBS, 0.43; YRI, 0.12; and CHB, 0.11 (*P* < 0.001); JPT, 0.06 (*P* = 0.02); and MEZs, 0.15 (*P* < 0.001)]. On the other hand, the frequencies of the synonymous alleles rs1042717A, rs1042718A, and rs1042719C were higher among MAs than in the other populations (*P* < 0.05) with the exception of JPT (Table 1). Importantly, the allele frequencies of rs1042717A and rs1042718A were significantly higher among MAs than in MXL or MEZs. In contrast, the frequency of the rs1042719C allele was similar between the MA and MXL groups, although it significantly differed between MAs and MEZs (Table 1).

The frequencies of all *ADRB2* alleles in the MEZ group were significantly different from those in the CEU and IBS populations, with the exception of rs1042713A in IBS. As expected, none of the variants frequencies significantly differed between MEZs and MXL. Similar to the findings in MAs, all of the variants (except rs1042714) exhibited very similar behavior in MEZs and JPT, but not in YRI and CHB (Table 1). Notably, the frequencies of all of the analyzed variants in the MEZ population exhibited an intermediate relationship to those observed in their ancestral populations, with IBS on one side and MAs on the other.

**Fig 1:**
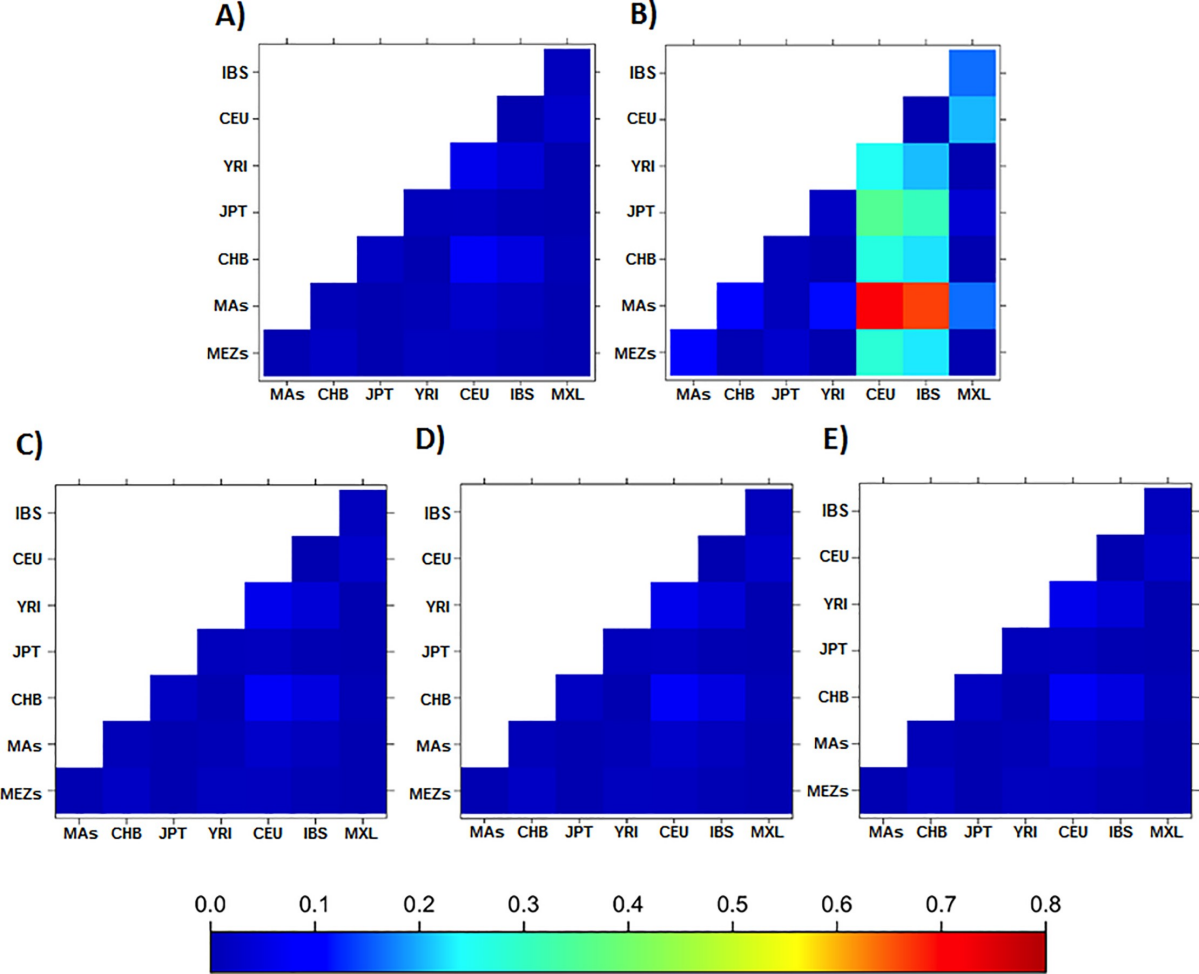
Level of genetic differentiation (*F*_ST_) for the five *ADRB2* variants among MAs, MEZs and other continental populations (see also S2 Table). Populations from the 1000 Genomes database were used. A) rs1042713A; B) rs1042714G; C) rs1042717A; D) rs1042718A; E) rs1042719C. The darkest blue indicates the lowest level of differentiation, whereas red indicates the highest *F*_ST_ value.

We also investigated the level of differentiation (*F*_ST_) of the five *ADRB2* polymorphisms between MAs, MEZs, and the CPs. The rs1042713 variant exhibited the lowest level of differentiation among all of the analyzed populations, whereas the rs1042714 variant exhibited the most extreme level of differentiation, particularly between MAs and CEU (0.709), and between MAs and IBS (0.665) (Fig 1 and S2 Table). With regards to rs1042717, rs1042718, and rs1042719, the highest levels of population differentiation were found between MAs and CEU (0.172, 0.185, and 0.125, respectively), and the lowest *F*_ST_ values were found between MAs and JPT (0.002, 0.000, and 0.000, respectively; Fig 1 and S2 Table).

**Fig 2:**
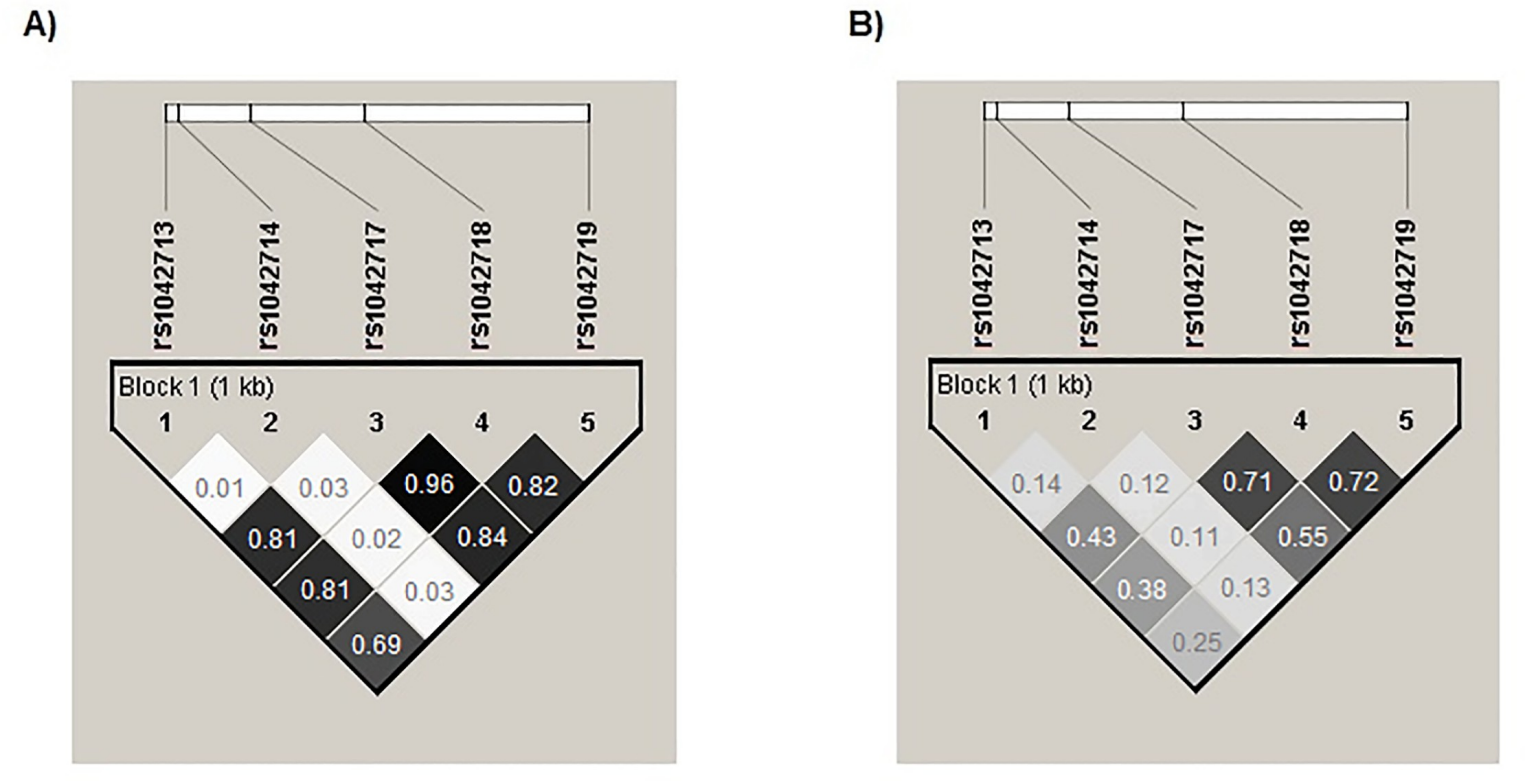
Linkage disequilibrium (LD) structure of *ADRB*2 SNPs in A) MAs and B) MEZs. The r^2^ value was calculated using Haploview software 4.2.

The synonymous variants rs1042717, rs1042718, and rs1042719 exhibited high LD in both the MA and MEZ populations, with higher values among MAs (r^2^ = 0.96, 0.84, and 0.82) than MEZs (r^2^ = 0.71, 0.55, and 0.72; Fig 2). In contrast, the non-synonymous SNP rs1042714 exhibited no evidence of LD with any other SNPs in either population (r^2^ = 0.01 to 0.03 among MAs, and r^2^ = 0.11 to 0.14 among MEZs; Fig 2). This analysis revealed five haplotypes with frequencies greater than 1%, of which four were shared by MEZs and MAs, as well as one haplotype with a low frequency (0.02) found only in MEZ individuals (Table 2). Interestingly, the frequencies of the four shared haplotypes significantly differed between the two groups (*P* < 0.001; Table 2).

**Table 2:** Haplotype frequencies of *ADRB2* SNPs in MA and MEZ populations.

Haplotypes		Frequencies		*P value*
		MEZs	MAs	
1	GCAAC	0.36	0.48	1x10^-8^
2	ACGCG	0.35	0.43	1x10^-8^
3	GGGCG	0.12	0.03	1x10^-8^
4	ACGCC	0.05	0.03	8x10^-5^
5	GCACC	0.02	NF	-

NF, not found

Since the three synonymous variants rs1042717, rs1042718, and rs1042719 showed a high LD in both MEZs and MAs, we only show the rs1042717 variant data from our further analyses.

### Allele frequencies of *ADRB2* variants among MA ethnic groups

We also compared the distribution of *ADRB2* variants between MAs belonging to different ethnic groups. This analysis included only Amerindian groups represented by at least 10 individuals which totaled 31 of the 62 ethnic groups (n = 1,851 individuals). We found that the *ADRB2* variants exhibited high heterogeneity among all of the ethnic groups. For example, the allele frequency of rs1042713A ranged from 0.23 in the Nahuatl group from Morelos to 0.72 in the Seri group, whereas that of rs1042717A ranged from 0.31 in Seris to 0.77 in Nahuatls from Morelos (Table 3). In contrast, the rs1042714G (Glu27) allele exhibited very low frequencies in all ethnic groups, with frequencies of <0.05 in 24 of the 31 analyzed groups, and frequencies of >0.10 among only the Purepechas and Mayos (0.107 and 0.125, respectively). This SNP was monomorphic in the Chuj, Huasteco, Huave, Kanjobal, Mocho, Tojolabal, and Nahuatl from Morelos ethnic groups (Table 3).

**Table 3:** Distribution of allele frequencies of *ADRB2* SNPs in MAs.

SNP	AF	SNP	AF	SNP	AF
rs1042713(G/A)	A	rs1042714(C/G)	G	Tag SNP rs1042717(G/A)	A
Nahuatl Mor^a^	0.23	Nahuatl Mor^a^	0.000	Seri^a^	0.31
Purepecha	0.29	Kanjobal^a^	0.000	Chontal Oax^a^	0.32
Kanjobal^a^	0.36	Huasteco	0.000	Pame	0.35
Zapoteco	0.40	Chuj	0.000	Jakalteko	0.36
Nahuatl Edo Mex	0.40	Mocho	0.000	Mam	0.38
Popoluca	0.41	Tojolabal	0.000	Mocho	0.40
Otomi	0.42	Huave	0.000	Nahuatl Pue	0.45
Nahuatl CDMX	0.43	Zapoteco	0.008	Mazateco	0.46
Nahuatl SLP	0.43	Mixteco	0.008	Mixteco	0.46
Maya	0.44	Nahuatl SLP	0.011	Chuj	0.47
Huasteco	0.45	Mam	0.012	Mayo	0.47
Mazahua	0.45	Kaqchikel	0.014	Yaqui	0.47
Kaqchikel	0.46	Chinanteco	0.019	Chinanteco	0.48
Totonaco	0.47	Tarahumara	0.022	Tarahumara	0.49
Yaqui	0.47	Totonaco	0.022	Mixe	0.50
Mixe	0.48	Mixe	0.024	Mazahua	0.50
Mayo	0.48	Nahuatl Edo Mex	0.024	Tojolabal	0.51
Chinanteco	0.49	Mazateco	0.025	Nahuatl CDMX	0.52
Huave	0.50	Otomi	0.026	Maya	0.53
Tojolabal	0.50	Seri^b^	0.026	Otomi	0.53
Mazateco	0.53	Yaqui	0.029	Totonaco	0.53
Mixteco	0.53	Nahuatl Pue	0.039	Popoluca	0.54
Nahuatl Pue	0.53	Jakalteko	0.040	Huave	0.55
Tarahumara	0.56	Maya	0.045	Nahuatl SLP	0.56
Mocho	0.57	Mazahua	0.050	Purepecha	0.57
Chuj	0.58	Pame	0.050	Nahuatl Edo Mex	0.57
Pame	0.60	Nahuatl CDMX	0.050	Huasteco	0.58
Mam	0.62	Popoluca	0.071	Kaqchikel	0.60
Chontal Oax^b^	0.62	Chontal Oax^b^	0.091	Zapoteco	0.60
Jakalteko	0.65	Purepecha	0.107	Kanjobal^b^	0.64
Seri^b^	0.72	Mayo	0.125	Nahuatl Mor^b^	0.77

a Ethnicities that showed the lowest frequency in all SNPs analyzed.

b Ethnicities that showed the highest frequency in all SNPs analyzed.

AF = allele frequencies

**Fig 3:**
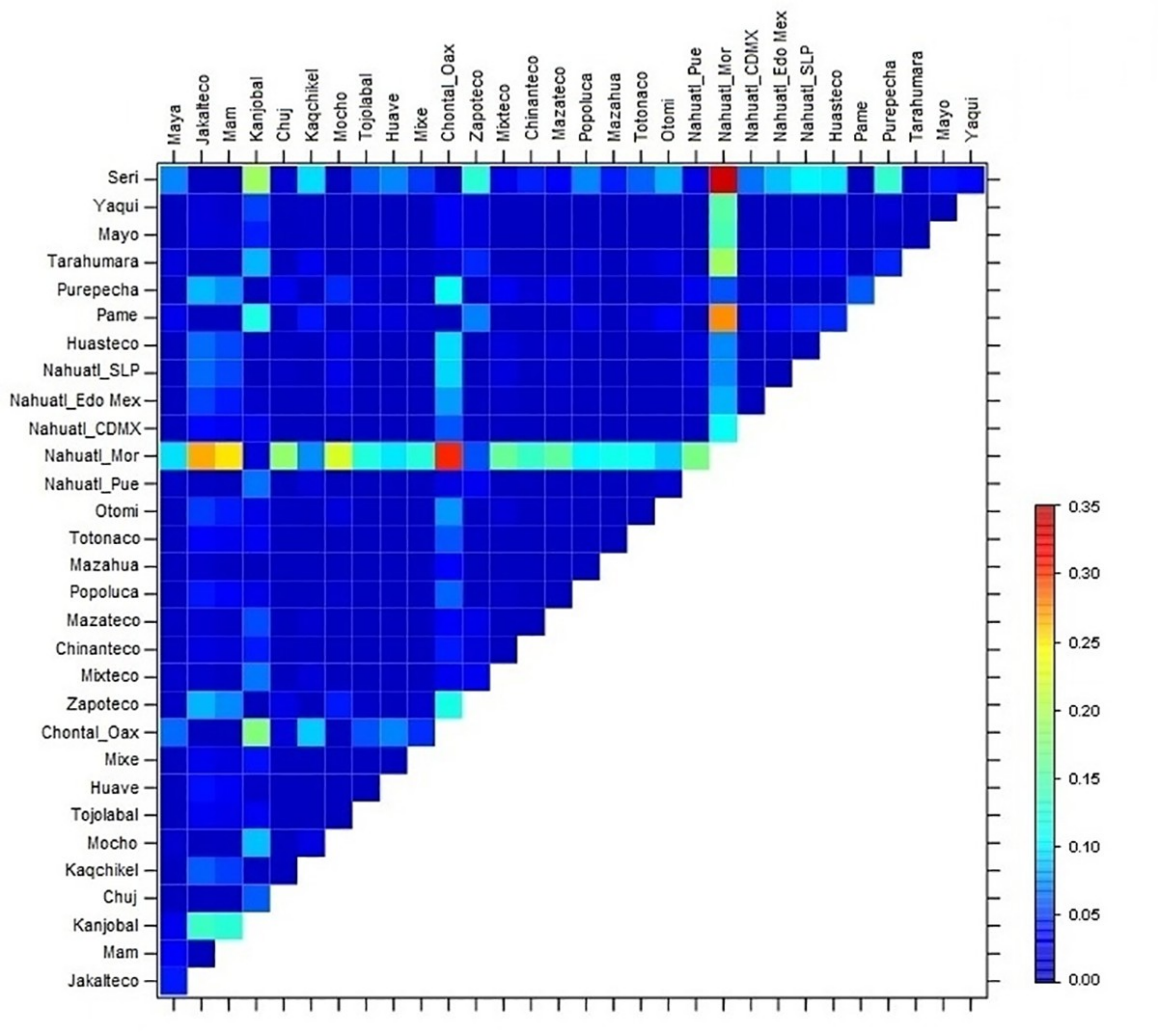
Pairwise *F*_ST_ values among the 31 MA ethnicities ordered geographically (see also S3 Table). Calculations were performed with the five *ADRB2* variants using GENEPOP software version 1.2. The darkest blue indicates the lowest level of differentiation, whereas red color indicates the highest *F*_ST_ value.

We determined the *F*_ST_ of the five *ADRB2* variants among 31 Amerindian groups, and found the highest level of differentiation in the Seri, Pame, Nahuatl from Morelos, Chontal, and Kanjobal groups (Fig 3). Notably, the Nahuatls from Morelos exhibited high population differentiation compared to most of the other groups (24 with *F*_ST_ > 0.10), but mainly compared to the Chontal and Pame (*F*_ST_ = 0.30 and 0.28, respectively). The Seri, Chontal, and Pame groups did not show any degree of differentiation between each other (*F*_ST_ = 0.00, 0.00, and 0.00, respectively; Fig 3, S3 Table).

### Geographic distribution of *ADRB2* variants among MA and MEZ individuals

**Fig 4:**
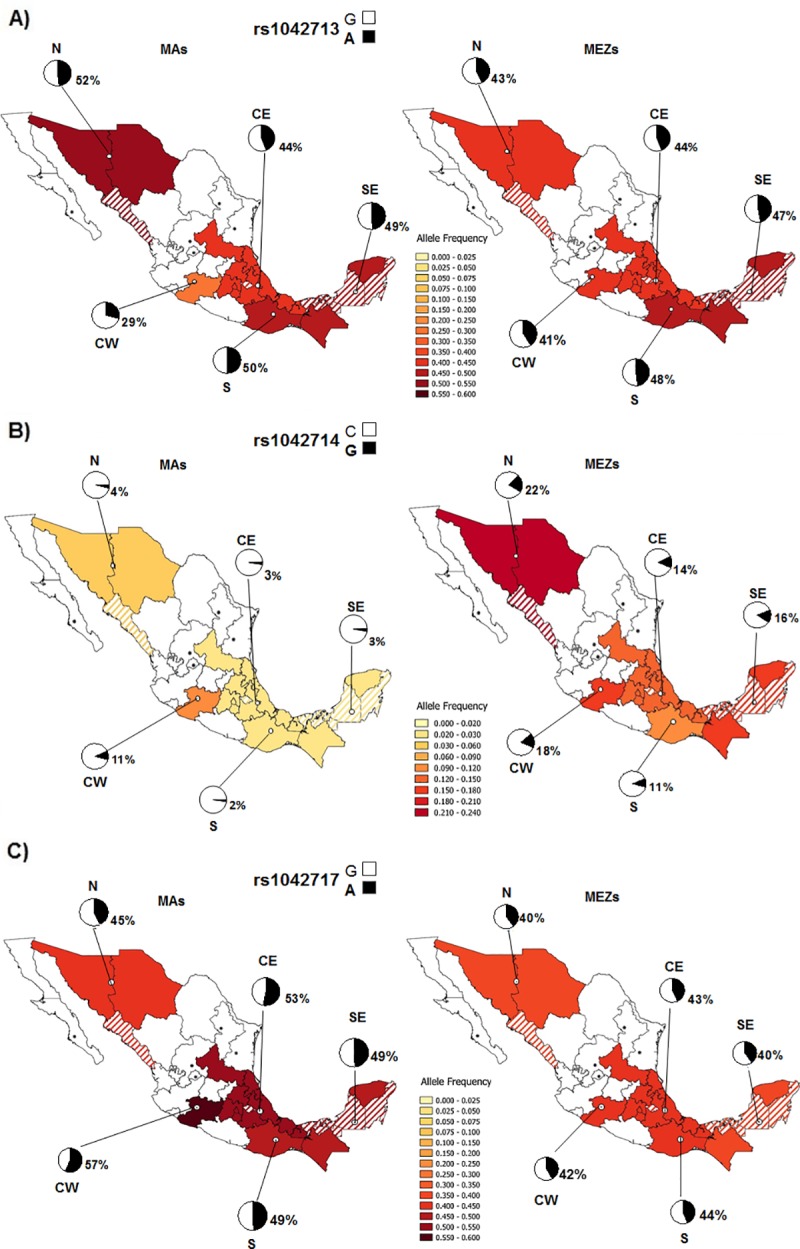
Geographic distribution of *ADRB2* alleles in five regions among MAs and MEZs. A) rs1042713A; B) rs1042714G; and C) tag SNP rs1042717A; North (N), Central East (CE), Central West (CW), South (S) and South East (SE). Striped States were not sampled because they are inhabited by neighboring indigenous included in this study. *States without indigenous population.

We sorted MA and MEZ individuals into five geographic regions, and found that the Seris in the North, Pames and Nahuatls from Morelos in the Central East, Chontals from the South, and Kanjobals from the South East exhibited extreme frequencies compared to other geographically close groups. Therefore, we removed these ethnic groups from the geographic analysis (Table 4). The geographic distribution of the rs1042713 variant did not significantly differ between MEZ and MA individuals (Fig 4A). In contrast, the Glu27 (G) allele of rs1042714 exhibited a significantly lower frequency among MAs than MEZs in all regions except the Central West region (Fig 4B). Interestingly, within the MEZ group, the frequency of rs1042714G decreased from 22% in the North to 11% in the South; whereas MAs exhibited a similar distribution of this allele in all regions except the Central West (Fig 4B). Similarly, the geographic distribution of the rs1042717 SNP did not significantly differ between the MEZ and MA populations, except for in the Central East region (Fig 4C).

**Table 4:** Geographic distribution and comparison of allele frequencies of *ADRB2* SNPs in 31 MA ethnic groups and the MEZ population.

Geographic Region	Ethnic Group (n)	rs1042713 A			rs1042714 G			rs1042717 A		
		AF	*P* *value*	*P* value*	AF	*P value*	*P* value*	AF	*P* *value*	*P* value*
**North**	Mayo (29)	0.483	0.38	NS	0.125	3x10^-3^^b^	0.01	0.466	0.87	NS
	Seri^a^ (19)	0.722			0.026			0.306		
	Tarahumara (93)	0.557			0.022			0.439		
	Yaqui (37)	0.472			0.029			0.472		
	**MAs** (159)	0.520	0.04^b^	NS	0.040	1x10^-8^^b^	1x10^-8^^b^	0.450	0.26	NS
	**MEZs** (122)	0.430			0.220^c^			0.400		
**Central West**	**MAs** [Purepecha (14)]	0.286	0.19	NS	0.107	0.32	NS	0.571	0.12	NS
	**MEZs** (180)	0.410			0.180^c^			0.420		
**Central East**	Huasteco (79)	0.447	0.79	NS	0.000	0.09	NS	0.576	0.8	NS
	Mazahua (10)	0.450			0.050			0.500		
	Nahuatl CDMX (53)	0.431			0.050			0.520		
	Nahuatl Edo Mex (22)	0.405			0.024			0.568		
	Nahuatl Mor^a^ (45)	0.227			0.000			0.773		
	Nahuatl Pue (52)	0.529			0.039			0.451		
	Nahuatl SLP (44)	0.430			0.011			0.558		
	Otomi (223)	0.424			0.026			0.533		
	Pame^a^(10)	0.600			0.050			0.350		
	Popoluca (36)	0.412			0.071			0.543		
	Totonaco (97)	0.468			0.022			0.527		
	**MAs** (616)	0.440	0.72	NS	0.027	1x10^-8^^b^	1x10^-8^^b^	0.530	1x10^-8^^b^	1x10^-8^^b^
	**MEZs** (1435)	0.440			0.140^c^			0.430		
**South**	Chinanteco (83)	0.494	0.31	NS	0.019	0.57	NS	0.475	0.15	NS
	Chontal Oax^a^ (44)	0.622			0.091			0.429		
	Huave (26)	0.500			0.000			0.477		
	Mazateco (61)	0.526			0.025			0.458		
	Mixe (90)	0.481			0.030			0.500		
	Mixteco (137)	0.534			0.008			0.459		
	Zapoteco (66)	0.396			0.008			0.600		
	**MAs** (463)	0.500	0.50	NS	0.015	1x10^-8^^b^	1x10^-8^^b^	0.490	0.14	NS
	**MEZs** (180)	0.480			0.110^c^			0.440		
**South East**	Chuj (17)	0.577	6x10^-3^^b^	NS	0.000	0.13	NS	0.469	0.01^b^	NS
	Jakalteko (40)	0.647			0.039			0.363		
	Kanjobal^a^ (29)	0.362			0.000			0.643		
	Kaqchikel (37)	0.457			0.014			0.600		
	Mam (45)	0.615			0.012			0.377		
	Maya (252)	0.437			0.045			0.526		
	Mocho (15)	0.571			0.000			0.400		
	Tojolabal (46)	0.500			0.000			0.511		
	**MAs** (452)	0.490	0.62	NS	0.030	1x10^-8^^b^	1x10^-8^^b^	0.490	0.05	NS
	**MEZs** (63)	0.470			0.160			0.400		
**Total**	**MAs** (1704)	0.480	2x10^-3^^b^	0.01	0.030	1x10^-8^^b^	1x10^-8^^b^	0.500	1x10^-8^^b^	1x10^-8^^b^
	**MEZs** (1980)	0.440			0.150			0.430		

a Ethnicities with divergent frequencies; not considered in the comparison.

b P values show the regions having significant differences.

c Decreased frequency from the North to South.

*P values (<0.05) after Bonferroni correction.

AF = Allele frequency

We investigated whether the geographic parameters of latitude, longitude and altitude might influence the distribution of the variants analyzed in this study. Our results indicated that only the frequency of the Glu27 (G) allele of rs1042714 showed a tendency of a significant negative correlation with longitude (*P* = 0.05), exhibiting a decreasing frequency from West to East.

## Discussion

After the initial out-of-Africa expansion, the combination of human long-range migration, genetic adaptations to changing environments, and admixture have led to great differences in the genetic structures of human groups with different ancestries [16,31–33]. Several studies demonstrate how these genetic differences influence people’s susceptibility to developing a diversity of chronic diseases, generating potential group-specific genetic risk factors [34]. It has been proposed that ancestral variants that conferred selection advantages during the early development of human populations may become maladaptive under current environmental conditions [35]. Thus, human geneticists are performing detailed investigations of the geographic distribution of genetic variations, enabling reevaluation of current models of peopling through the world, and of the importance of natural selection in determining the geographic distribution of phenotypes [33].

In the present study, we investigated frequency distributions of the alleles and genotypes of five risk-associated SNPs located in the coding region of *ADRB2* within both MEZ and MA groups. Our analyses revealed great diversity in the frequency distributions of the individual variants, not only between MEZs and MAs, but also among the different studied MA groups.

Among the five analyzed variants, the Glu27 (G) allele of rs1042714G exhibited the greatest differences in frequency between MAs and other populations worldwide. MA individuals showed the lowest frequency of this G allele, reported to date. In fact, this allele was absent in most of the analyzed MA groups, with frequencies of >0.05 in only four ethnic groups (Popoluca, Chontal Oax, Purepecha, and Mayo). These frequencies were still significantly lower than those observed among MEZs (0.15) or IBS (0.43), an ancestral population of MEZ individuals. These findings are relevant because this variant, which has a Gln substituted for Glu at position 27 in the protein, shows strong association with a variety of chronic degenerative diseases, including asthma, obesity, coronary artery disease, myocardial infarction, type 2 diabetes and, more recently, with longevity and acclimatization [10,14,36–38].

Notably, the Gln27 (C) allele of rs1042714 has been considered an energy-expense allele, which may protect humans from extreme temperature changes [18]. Thus, the high frequency of Gln27 in MA individuals (97%) may have resulted from selection pressures due to extreme low temperatures during the glacial period before humans emigrated from Beringia, which worked against the ancestral Glu27 allele and favored selection of the derived Gln27 allele. The almost exclusive presence of the energy-expense Gln27 allele in the MA population similar to observations in the Japanese and Han Chinese populations (94.2% and 95.2%, respectively) supports the notion that MAs may be descendants from groups that came from East Asia, which were subjected to extreme low temperatures during the glacial period. It has been hypothesized that human dispersion in northeast Asia immediately before and after the Last Glacial Maximum most likely led to the settlement of Beringia, and ultimately of the Americas [39,40]. Similar observations have been reported with the variants -217A, 825T, and -246G in the *AGT*, *GNB3*, and *ENaCα* genes, respectively, which are associated with hypertension. This differential susceptibility may be due to exposure to selection pressures during human adaptation to climate change [16].

We also found that the Nahuatl from Morelos exhibited the most divergent frequencies of the five *ADRB2* markers, followed by the Seris. Previous reports have described the high level of differentiation within the Seri group [3, 4], but not the high differentiation in the Nahuatl from Morelos group. Importantly, we found a high degree of population differentiation between the Nahuatl from Morelos in the Central East region and Seris from the North (0.341). Using these five SNPs, differences were higher than that previously observed between the CEU and CHB populations from the 1000 Genomes (0.108). These findings support the previously reported by Moreno et al., who by using a genome wide scan technology, also observed a higher differentiation between some Mexican Amerindian groups (Seris and Lacandon: 0.136) than that found between CEU and CHB populations (0.11) [4,41].

On limitation of this study is that we analyzed only five markers in a single gene. However the high differentiation observed among the different Amerindian ethnic groups may still be interpreted as indicating several possible events: 1) the settlement of new colonies by founder effects; 2) the presence of strong “bottlenecks”; 3) positive selection for alleles that were appropriate in the new environments; and 4) increased allele frequency due to allele surfing, a process in which a small subset of individuals expands and multiplies into an unsettled territory. Despite the highly diverse frequencies of the analyzed SNPs, we identified the same types of haplotypes among MAs and MEZs, with the exception of one low-frequency haplotype that was found only among MEZ individuals. This behavior was most likely due to the high LD observed between the three synonymous SNPs.

Of the five analyzed variants, rs1042714 was the only one to show a geographic gradient across the Mexican territory, with a decreasing frequency from North to South, among MEZ individuals but not among MAs. It is well known that MA individuals have contributed along with Caucasian and, to a lesser extent, African individual towards the generation of the current MEZ population in Mexico, with a gradient of Caucasian ancestry decreasing from North to South [3,4]. Many reports of European, African, and Asian populations suggest that *ADRB2* has been subjected to either balancing selection or a selective sweep [17,18]. However, the Glu27 (G) allele of rs1042714, which is almost absent among MAs but carried at a high frequency among Europeans, may have been enriched in our population at the time that Spanish people colonized Mexico. Notably, Gorlov et al. [42] tested the hypothesis that SNPs that influence disease risk undergo positive or negative selection more frequently than the average SNP in the human genome. They suggested that diseases play a central role in human evolution, directly or indirectly influencing the population frequencies of genetic variants via hitchhiking or bottleneck effects.

In conclusion, the Gln27Glu variant in *ADRB2*, which is associated with a broad range of phenotypes, is an excellent paragon supporting Gorlov’s hypothesis that risk alleles may be susceptible to different selection pressures. The extreme low temperatures during the glacial period could have worked against the ancestral Glu27 allele, which suffered negative selection in groups that came from East Asia and settled the Americas, while Gln27, an energy-expense allele that may protect humans from extreme temperature changes, experienced positive selection. Moreover, our results showed that the majority of Glu27 alleles in the MEZ population seemed to be an exclusively Caucasian contribution. Consequently, in the MEZ population, the phenotypes associated with this variant could have a Caucasian heritage, whereas the traits associated with Gln27 may have a predominantly Amerindian contribution. Similar to *ADRB2*, other disease susceptibility genes may also undergo selection pressure. This kind of study is critical for understanding the importance of assessing the population structure and analyzing the behaviors of the genetic components of populations that harbor great diversity, such as MAs, which may contribute and influence biomedical traits in the MEZ population. The present in depth analysis of *ADRB2* variants and haplotypes among MAs and MEZs improves our understanding of ethnic and individual differences in the contribution of *ADRB2* to disease susceptibility within the Mexican population.

## Supporting information

S1 Table Geographic distribution of genotype *ADRB2* SNPs. Geographic distribution of genotype frequencies of *ADRB2* SNPs among 31 Mexican Amerindians (MAs) Ethnic Groups and Mexican Mestizos (MEZs).
(DOC)

S2 Table *F*_ST_ values among MEZs, MAs and CPs. *F*_ST_ values among Mexican Mestizos (MEZs), Mexican Amerindians (MAs), and five continental populations (CPs) for the *ADRB2* variants analyzed in this study.
(DOC)

S3 Table *F*_ST_ values among Mexican Amerindian groups. *F*_ST_ values for the *ADRB2* variants; rs1042713A, rs1042714G rs1042717A, rs1042718A and rs1042719C among the Mexican Amerindian groups sorted by geographic region.
(DOC)
