# Supplementary material for: Genetic variability of five ADRB2 polymorphisms among Mexican Amerindian ethnicities and the Mestizo population
Source: PLoS One. 2019 Dec 2;14(12):e0225030. doi: 10.1371/journal.pone.0225030 (PMC6886845; doi:10.1371/journal.pone.0225030)
Supplement: S1 Table — Geographic distribution of genotype frequencies of ADRB2 SNPs among 31 Mexican Amerindians (MAs) Ethnic Groups and Mexican Mestizos (MEZs). (DOC) [file pone.0225030.s001.doc]

**S1 Table. Geographic distribution of genotype frequencies of *ADRB2* SNPs among 31 Mexican Amerindians (MAs) Ethnic Groups and Mexican Mestizos (MEZs).**

| **Geographic** | **Ethnic Group (n)** | **rs1042713** | | |  | **rs1042714** | | |  | **rs1042717** | | |  | **rs1042718** | | |  | **rs1042719** | | |
| --- | --- | --- | --- | --- | --- | --- | --- | --- | --- | --- | --- | --- | --- | --- | --- | --- | --- | --- | --- | --- |
| **Region** |  | **GG** | **GA** | **AA** |  | **CC** | **CG** | **GG** |  | **GG** | **GA** | **AA** |  | **CC** | **CA** | **AA** |  | **GG** | **GC** | **CC** |
| **North** | **Mayo (29)** | 0.310 | 0.414 | 0.276 |  | 0.750 | 0.250 | 0.000 |  | 0.310 | 0.448 | 0.241 |  | 0.310 | 0.448 | 0.241 |  | 0.250 | 0.429 | 0.321 |
|  | **Seria (19)** | 0.111 | 0.333 | 0.556 |  | 0.947 | 0.053 | 0.000 |  | 0.500 | 0.389 | 0.111 |  | 0.474 | 0.421 | 0.105 |  | 0.500 | 0.389 | 0.111 |
|  | **Tarahumara (93)** | 0.227 | 0.432 | 0.341 |  | 0.957 | 0.043 | 0.000 |  | 0.356 | 0.411 | 0.233 |  | 0.391 | 0.437 | 0.172 |  | 0.297 | 0.451 | 0.253 |
|  | **Yaqui (37)** | 0.278 | 0.500 | 0.222 |  | 0.943 | 0.057 | 0.000 |  | 0.250 | 0.556 | 0.194 |  | 0.243 | 0.568 | 0.189 |  | 0.250 | 0.556 | 0.194 |
|  | **MAs Total (159)** | 0.255 | 0.444 | 0.301 |  | 0.916 | 0.084 | 0.000 |  | 0.323 | 0.452 | 0.226 |  | 0.340 | 0.471 | 0.190 |  | 0.277 | 0.471 | 0.252 |
|  | **MEZs Total (122)** | 0.336 | 0.462 | 0.202 |  | 0.587 | 0.394 | 0.018 |  | 0.353 | 0.487 | 0.160 |  | 0.437 | 0.420 | 0.143 |  | 0.308 | 0.542 | 0.150 |
| **Center** | **MAs [Purepecha (14)]** | 0.429 | 0.571 | 0.000 |  | 0.786 | 0.214 | 0.000 |  | 0.143 | 0.571 | 0.286 |  | 0.143 | 0.571 | 0.286 |  | 0.154 | 0.538 | 0.308 |
| **West** | **MEZs(180)** | 0.351 | 0.476 | 0.173 |  | 0.670 | 0.295 | 0.034 |  | 0.357 | 0.450 | 0.193 |  | 0.349 | 0.471 | 0.180 |  | 0.289 | 0.497 | 0.214 |
| **Center**  **East** | **Huasteco (79)** | 0.400 | 0.307 | 0.293 |  | 1.000 | 0.000 | 0.000 |  | 0.215 | 0.418 | 0.367 |  | 0.215 | 0.418 | 0.367 |  | 0.203 | 0.418 | 0.380 |
|  | **Mazahua (10)** | 0.300 | 0.500 | 0.200 |  | 0.900 | 0.100 | 0.000 |  | 0.300 | 0.400 | 0.300 |  | 0.300 | 0.400 | 0.300 |  | 0.200 | 0.500 | 0.300 |
|  | **Nahuatl CDMX (53)** | 0.314 | 0.510 | 0.176 |  | 0.900 | 0.100 | 0.000 |  | 0.235 | 0.490 | 0.275 |  | 0.269 | 0.442 | 0.288 |  | 0.204 | 0.531 | 0.265 |
|  | **Nahuatl EdoM (22)** | 0.333 | 0.524 | 0.143 |  | 0.952 | 0.048 | 0.000 |  | 0.182 | 0.500 | 0.318 |  | 0.182 | 0.500 | 0.318 |  | 0.227 | 0.500 | 0.273 |
|  | **Nahuatl Mora (45)** | 0.591 | 0.364 | 0.045 |  | 1.000 | 0.000 | 0.000 |  | 0.045 | 0.364 | 0.591 |  | 0.044 | 0.378 | 0.578 |  | 0.049 | 0.341 | 0.610 |
|  | **Nahuatl Pue (52)** | 0.196 | 0.549 | 0.255 |  | 0.922 | 0.078 | 0.000 |  | 0.294 | 0.510 | 0.196 |  | 0.300 | 0.500 | 0.200 |  | 0.255 | 0.532 | 0.213 |
|  | **Nahuatl SLP (44)** | 0.326 | 0.488 | 0.186 |  | 0.977 | 0.023 | 0.000 |  | 0.186 | 0.512 | 0.302 |  | 0.186 | 0.512 | 0.302 |  | 0.175 | 0.425 | 0.400 |
|  | **Otomi (223)** | 0.298 | 0.555 | 0.147 |  | 0.949 | 0.051 | 0.000 |  | 0.181 | 0.572 | 0.247 |  | 0.190 | 0.575 | 0.235 |  | 0.157 | 0.556 | 0.287 |
|  | **Pamea (10)** | 0.200 | 0.400 | 0.400 |  | 0.900 | 0.100 | 0.000 |  | 0.500 | 0.300 | 0.200 |  | 0.500 | 0.300 | 0.200 |  | 0.500 | 0.200 | 0.300 |
|  | **Popoluca (36)** | 0.353 | 0.471 | 0.176 |  | 0.857 | 0.143 | 0.000 |  | 0.171 | 0.571 | 0.257 |  | 0.273 | 0.455 | 0.273 |  | 0.171 | 0.600 | 0.229 |
|  | **Totonaco (97)** | 0.351 | 0.364 | 0.286 |  | 0.957 | 0.043 | 0.000 |  | 0.226 | 0.495 | 0.280 |  | 0.247 | 0.473 | 0.280 |  | 0.234 | 0.479 | 0.287 |
|  | **MAs Total (616)** | 0.317 | 0.481 | 0.202 |  | 0.946 | 0.054 | 0.000 |  | 0.209 | 0.518 | 0.274 |  | 0.224 | 0.504 | 0.272 |  | 0.193 | 0.512 | 0.296 |
|  | **MEZs Total (1435)** | 0.323 | 0.482 | 0.195 |  | 0.739 | 0.240 | 0.021 |  | 0.315 | 0.504 | 0.181 |  | 0.336 | 0.481 | 0.183 |  | 0.269 | 0.503 | 0.229 |
| **South** | **Chinanteco (83)** | 0.288 | 0.438 | 0.275 |  | 0.962 | 0.038 | 0.000 |  | 0.309 | 0.432 | 0.259 |  | 0.296 | 0.444 | 0.259 |  | 0.250 | 0.438 | 0.313 |
|  | **Chontal_Oaxa (44)** | 0.122 | 0.512 | 0.366 |  | 0.818 | 0.182 | 0.000 |  | 0.286 | 0.571 | 0.143 |  | 0.465 | 0.419 | 0.116 |  | 0.465 | 0.395 | 0.140 |
|  | **Huave (26)** | 0.308 | 0.385 | 0.308 |  | 1.000 | 0.000 | 0.000 |  | 0.318 | 0.409 | 0.273 |  | 0.269 | 0.385 | 0.346 |  | 0.208 | 0.375 | 0.417 |
|  | **Mazateco (61)** | 0.190 | 0.569 | 0.241 |  | 0.951 | 0.049 | 0.000 |  | 0.237 | 0.610 | 0.153 |  | 0.237 | 0.610 | 0.153 |  | 0.207 | 0.534 | 0.259 |
|  | **Mixe (90)** | 0.275 | 0.488 | 0.238 |  | 0.953 | 0.047 | 0.000 |  | 0.258 | 0.483 | 0.258 |  | 0.241 | 0.506 | 0.253 |  | 0.273 | 0.432 | 0.295 |
|  | **Mixteco (137)** | 0.252 | 0.427 | 0.321 |  | 0.984 | 0.016 | 0.000 |  | 0.328 | 0.425 | 0.246 |  | 0.301 | 0.451 | 0.248 |  | 0.301 | 0.466 | 0.233 |
|  | **Zapoteco (66)** | 0.354 | 0.500 | 0.146 |  | 0.984 | 0.016 | 0.000 |  | 0.138 | 0.523 | 0.338 |  | 0.138 | 0.523 | 0.338 |  | 0.143 | 0.524 | 0.333 |
|  | **MAs Total (463)** | 0.270 | 0.466 | 0.265 |  | 0.970 | 0.030 | 0.000 |  | 0.271 | 0.476 | 0.253 |  | 0.255 | 0.488 | 0.257 |  | 0.247 | 0.466 | 0.287 |
|  | **MEZs Total (180)** | 0.291 | 0.467 | 0.242 |  | 0.794 | 0.188 | 0.018 |  | 0.308 | 0.497 | 0.195 |  | 0.324 | 0.474 | 0.202 |  | 0.273 | 0.488 | 0.238 |
| **South**  **East** | **Chuj (17)** | 0.231 | 0.385 | 0.385 |  | 1.000 | 0.000 | 0.000 |  | 0.250 | 0.563 | 0.188 |  | 0.235 | 0.588 | 0.176 |  | 0.235 | 0.588 | 0.176 |
|  | **Jakalteko (40)** | 0.235 | 0.235 | 0.529 |  | 0.921 | 0.079 | 0.000 |  | 0.425 | 0.425 | 0.150 |  | 0.425 | 0.425 | 0.150 |  | 0.333 | 0.487 | 0.179 |
|  | **Kanjobala(29)** | 0.483 | 0.310 | 0.207 |  | 1.000 | 0.000 | 0.000 |  | 0.214 | 0.286 | 0.500 |  | 0.214 | 0.286 | 0.500 |  | 0.172 | 0.172 | 0.655 |
|  | **Kaqchikel (37)** | 0.371 | 0.343 | 0.286 |  | 0.973 | 0.027 | 0.000 |  | 0.200 | 0.400 | 0.400 |  | 0.222 | 0.389 | 0.389 |  | 0.243 | 0.378 | 0.378 |
|  | **Mam (45)** | 0.179 | 0.410 | 0.410 |  | 0.977 | 0.023 | 0.000 |  | 0.422 | 0.400 | 0.178 |  | 0.432 | 0.364 | 0.205 |  | 0.378 | 0.422 | 0.200 |
|  | **Maya (252)** | 0.312 | 0.502 | 0.186 |  | 0.911 | 0.089 | 0.000 |  | 0.214 | 0.521 | 0.265 |  | 0.226 | 0.523 | 0.251 |  | 0.196 | 0.515 | 0.289 |
|  | **Mocho (15)** | 0.143 | 0.571 | 0.286 |  | 1.000 | 0.000 | 0.000 |  | 0.333 | 0.533 | 0.133 |  | 0.267 | 0.600 | 0.133 |  | 0.286 | 0.643 | 0.071 |
|  | **Tojolabal (46)** | 0.217 | 0.565 | 0.217 |  | 1.000 | 0.000 | 0.000 |  | 0.217 | 0.543 | 0.239 |  | 0.227 | 0.545 | 0.227 |  | 0.159 | 0.545 | 0.295 |
|  | **MAs Total (463)** | 0.278 | 0.462 | 0.260 |  | 0.939 | 0.061 | 0.000 |  | 0.260 | 0.494 | 0.246 |  | 0.267 | 0.494 | 0.239 |  | 0.232 | 0.501 | 0.267 |
|  | **MEZs Total (63)** | 0.300 | 0.467 | 0.233 |  | 0.705 | 0.279 | 0.016 |  | 0.373 | 0.458 | 0.169 |  | 0.410 | 0.426 | 0.164 |  | 0.328 | 0.492 | 0.180 |
