# Supplementary material for: Genetic variability of five ADRB2 polymorphisms among Mexican Amerindian ethnicities and the Mestizo population
Source: PLoS One. 2019 Dec 2;14(12):e0225030. doi: 10.1371/journal.pone.0225030 (PMC6886845; doi:10.1371/journal.pone.0225030)
Supplement: S2 Table — FST values among Mexican Mestizos (MEZs), Mexican Amerindians (MAs), and five continental populations (CPs) for the ADRB2 variants analyzed in this study. (DOC) [file pone.0225030.s002.doc]

**S2 Table.** *F*_ST_ Values among Mexican Mestizos (MEZs), Mexican Amerindians (MAs), and Five Continental Populations for the *ADRB2* Variants Analyzed in This Study.

|  | **rs1042713** | | | | | | | |  | **rs1042714** | | | | | | | |
| --- | --- | --- | --- | --- | --- | --- | --- | --- | --- | --- | --- | --- | --- | --- | --- | --- | --- |
|  | **MEZs** | **MAs** | **CHB** | **JPT** | **YRI** | **CEU** | **IBS** | **MXL** |  | **MEZs** | **MAs** | **CHB** | **JPT** | **YRI** | **CEU** | **IBS** | **MXL** |
| **MXL** | 0.000 | 0.000 | 0.005 | 0.000 | 0.000 | 0.028 | 0.012 | - |  | 0.000 | 0.164 | 0.000 | 0.035 | 0.000 | 0.204 | 0.165 | - |
| **IBS** | 0.004 | ***0.013*** | 0.050 | 0.003 | 0.039 | 0.000 | - | 0.012 |  | 0.229 | ***0.665*** | 0.225 | 0.306 | 0.206 | 0.000 | - | 0.165 |
| **CEU** | 0.013 | ***0.027*** | 0.074 | 0.014 | 0.062 | - | 0.000 | 0.028 |  | 0.279 | ***0.709*** | 0.269 | 0.354 | 0.250 | - | 0.000 | 0.204 |
| **YRI** | 0.015 | 0.005 | 0.000 | 0.011 | - | 0.062 | 0.039 | 0.000 |  | 0.000 | 0.113 | 0.000 | 0.019 | - | 0.250 | 0.206 | 0.000 |
| **JPT** | **0.000** | **0.000** | 0.018 | - | 0.011 | 0.014 | 0.003 | 0.000 |  | **0.029** | **0.011** | 0.011 | - | 0.019 | 0.354 | 0.306 | 0.035 |
| **CHB** | 0.022 | 0.009 | - | 0.018 | 0.000 | 0.074 | 0.050 | 0.005 |  | 0.004 | 0.085 | - | 0.011 | 0.000 | 0.269 | 0.225 | 0.000 |
| **MAs** | **0.002** | - | 0.009 | **0.000** | 0.005 | ***0.027*** | ***0.013*** | 0.000 |  | **0.082** | - | 0.085 | **0.011** | 0.113 | ***0.709*** | ***0.665*** | 0.164 |
| **MEZ** | **-** | **0.002** | 0.022 | **0.000** | 0.015 | 0.013 | 0.004 | 0.000 |  | **-** | **0.082** | 0.004 | **0.029** | 0.000 | 0.279 | 0.229 | 0.000 |

|  | **Tag SNP rs1042717** | | | | | | | |  | **rs1042718** | | | | | | | |  | | **rs1042719** | | | | | | | |
| --- | --- | --- | --- | --- | --- | --- | --- | --- | --- | --- | --- | --- | --- | --- | --- | --- | --- | --- | --- | --- | --- | --- | --- | --- | --- | --- | --- |
|  | **MEZs** | **MAs** | **CHB** | **JPT** | **YRI** | **CEU** | **IBS** | **MXL** |  | **MEZs** | **MAs** | **CHB** | **JPT** | **YRI** | **CEU** | **IBS** | **MXL** |  | | **MEZs** | **MAs** | **CHB** | **JPT** | **YRI** | **CEU** | **IBS** | **MXL** |
| **MXL** | 0.002 | 0.030 | 0.000 | 0.009 | 0.000 | 0.081 | 0.083 | - |  | 0.000 | 0.028 | 0.000 | 0.010 | 0.000 | 0.104 | 0.127 | - |  | 0.000 | | 0.000 | 0.000 | 0.000 | 0.034 | 0.103 | 0.088 | - |
| **IBS** | 0.107 | ***0.172*** | 0.051 | 0.155 | 0.059 | 0.000 | - | 0.083 |  | 0.131 | ***0.202*** | 0.093 | 0.207 | 0.091 | 0.000 | - | 0.13 |  | 0.068 | | ***0.110*** | 0.051 | 0.129 | 0.007 | 0.000 | - | 0.09 |
| **CEU** | 0.107 | ***0.172*** | 0.050 | 0.153 | 0.06 | - | 0.000 | 0.081 |  | 0.116 | ***0.185*** | 0.075 | 0.182 | 0.072 | - | 0.000 | 0.104 |  | 0.080 | | ***0.125*** | 0.063 | 0.146 | 0.013 | - | 0.000 | 0.103 |
| **YRI** | 0.011 | 0.047 | 0.000 | 0.022 | - | 0.058 | 0.059 | 0.000 |  | 0.009 | 0.047 | 0.000 | 0.026 | - | 0.072 | 0.091 | 0.000 |  | 0.027 | | 0.058 | 0.012 | 0.067 | - | 0.013 | 0.007 | 0.034 |
| **JPT** | **0.000** | **0.002** | 0.028 | - | 0.022 | 0.153 | 0.155 | 0.009 |  | **0.002** | **0.000** | 0.025 | - | 0.026 | 0.182 | 0.207 | 0.010 |  | **0.007** | | **0.000** | 0.016 | - | 0.067 | 0.146 | 0.129 | 0.000 |
| **CHB** | 0.016 | 0.054 | - | 0.028 | 0.000 | 0.050 | 0.051 | 0.000 |  | 0.009 | 0.045 | - | 0.025 | 0.000 | 0.075 | 0.093 | 0.000 |  | 0.000 | | 0.013 | - | 0.016 | 0.012 | 0.063 | 0.051 | 0.000 |
| **MAs** | **0.012** | - | 0.054 | **0.002** | 0.047 | ***0.172*** | ***0.172*** | 0.03 |  | **0.014** | - | 0.045 | **0.000** | 0.047 | ***0.185*** | ***0.202*** | 0.028 |  | **0.006** | | - | 0.013 | **0.000** | 0.058 | ***0.125*** | ***0.110*** | 0.000 |
| **MEZ** | **-** | **0.012** | 0.016 | **0.000** | 0.011 | 0.107 | 0.107 | 0.002 |  | **-** | **0.014** | 0.009 | **0.002** | 0.009 | 0.116 | 0.131 | 0.000 |  | **-** | | **0.006** | 0.000 | **0.007** | 0.027 | 0.080 | 0.068 | 0.000 |

Abbreviations: CEU, Utah Residents (CEPH) with Northern and Western European Ancestry; CHB, Han Chinese in Beijing, China; IBS, Iberian Population in Spain; JPT, Japanese in Tokyo, Japan; MAs, Mexican Amerindians; MEZs, Mexican Mestizos; MXL, Mexican Ancestry from Los Angeles USA; SNP, single nucleotide polymorphism; YRI, Yoruba in Ibadan, Nigeria.

Pairwise *F*_ST_ statistics between Mexican Mestizos, Mexican Amerindians and 1000 genomes populations. Calculations were performed with GENEPOP software version 1.2 for each SNP. Estimates are based on genotypes and allele frequencies of each one of them.

Bold values show the *F*_ST_ level among MAs and MEZs.

Bold values in shaded boxes highlight the *F*_ST_ values observed between both Mexican population with JPT.

Italic values in bold, indicate the levels of population differentiation between MAs vs IBS and MAs vs CEU.
