# Supplementary material for: Genetic variability of five ADRB2 polymorphisms among Mexican Amerindian ethnicities and the Mestizo population
Source: PLoS One. 2019 Dec 2;14(12):e0225030. doi: 10.1371/journal.pone.0225030 (PMC6886845; doi:10.1371/journal.pone.0225030)
Supplement: S3 Table — FST values for the ADRB2 variants; rs1042713A, rs1042714G rs1042717A, rs1042718A and rs1042719C among the Mexican Amerindian groups sorted by geographic region. (DOC) [file pone.0225030.s003.doc]

**S3 Table.** *F*_ST_ Values for the *ADRB2* Variants; rs1042713A, rs1042714G rs1042717A, rs1042718A and rs1042719C Among the Mexican Amerindian Groups Sorted by Geographic Region.

| **Region** | | **SE** | | | | | | | | **S** | | | | | | | **CE** | | | | | | | | | | | **CW** | **N** | | | | | | |
| --- | --- | --- | --- | --- | --- | --- | --- | --- | --- | --- | --- | --- | --- | --- | --- | --- | --- | --- | --- | --- | --- | --- | --- | --- | --- | --- | --- | --- | --- | --- | --- | --- | --- | --- | --- |
| **Region** | **Ethnic groups** | **Maya** | **Jakalteko** | **Mam** | **Kanjobal** | **Chuj** | **Kaqchikel** | **Mocho** | **Tojolabal** | **Huave** | **Mixe** | **Chontal_Oax** | **Zapoteco** | **Mixteco** | **Chinanteco** | **Mazateco** | **Popoluca** | **Mazahua** | **Totonaco** | **Otomi** | **Nahuatl_Pue** | **Nahuatl_Mor** | **Nahuatl_CDMX** | **Nahuatl_Edo Mex** | **Nahuatl_SLP** | **Huasteco** | **Pame** | **Purepecha** | **Tarahuma** | **Mayo** | **Yaqui** | **Seri** | | |  |
| **N** | **Seri** | 0.07 | 0.00 | 0.00 | **0.19** | 0.01 | **0.10** | 0.00 | 0.06 | 0.07 | 0.05 | **0.00** | **0.13** | 0.03 | 0.04 | 0.03 | 0.07 | 0.04 | 0.06 | 0.09 | 0.02 | **0.34** | 0.07 | 0.09 | **0.10** | **0.10** | 0.00 | **0.13** | 0.01 | 0.04 | 0.04 | - |  |  | |
|  | **Yaqui** | 0.00 | 0.01 | 0.01 | 0.05 | 0.00 | 0.00 | 0.00 | 0.00 | 0.00 | 0.00 | 0.03 | 0.02 | 0.00 | 0.00 | 0.00 | 0.00 | 0.00 | 0.00 | 0.00 | 0.00 | **0.15** | 0.00 | 0.00 | 0.01 | 0.01 | 0.00 | 0.01 | 0.00 | 0.00 | - |  |  |  | |
|  | **Mayo** | 0.00 | 0.01 | 0.01 | 0.04 | 0.00 | 0.00 | 0.00 | 0.00 | 0.00 | 0.00 | 0.03 | 0.02 | 0.00 | 0.00 | 0.00 | 0.00 | 0.00 | 0.00 | 0.00 | 0.00 | **0.15** | 0.00 | 0.00 | 0.00 | 0.01 | 0.00 | 0.00 | 0.00 | - |  |  | | |  |
|  | **Tarahuma** | 0.02 | 0.00 | 0.00 | 0.09 | 0.00 | 0.03 | 0.00 | 0.01 | 0.01 | 0.01 | 0.01 | 0.05 | 0.00 | 0.00 | 0.00 | 0.01 | 0.00 | 0.01 | 0.02 | 0.00 | **0.19** | 0.01 | 0.02 | 0.03 | 0.03 | 0.00 | 0.05 | - |  |  |  | | |  |
| **CW** | **Purepecha** | 0.00 | 0.09 | 0.08 | 0.00 | 0.03 | 0.00 | 0.05 | 0.01 | 0.00 | 0.01 | **0.11** | 0.00 | 0.03 | 0.01 | 0.03 | 0.00 | 0.00 | 0.00 | 0.00 | 0.03 | 0.06 | 0.00 | 0.00 | 0.00 | 0.00 | 0.06 | - |  |  |  |  | | |  |
| **CE** | **Pame** | 0.02 | 0.00 | 0.00 | **0.12** | 0.00 | 0.04 | 0.00 | 0.01 | 0.02 | 0.01 | **0.00** | 0.07 | 0.00 | 0.00 | 0.00 | 0.02 | 0.00 | 0.01 | 0.04 | 0.00 | **0.28** | 0.02 | 0.03 | 0.05 | 0.05 | - |  |  |  |  |  | | |  |
|  | **Huasteco** | 0.00 | 0.07 | 0.06 | 0.01 | 0.00 | 0.00 | 0.02 | 0.00 | 0.00 | 0.00 | **0.10** | 0.00 | 0.02 | 0.01 | 0.01 | 0.00 | 0.00 | 0.00 | 0.00 | 0.02 | 0.07 | 0.00 | 0.00 | 0.00 | - |  |  |  |  |  |  | | |  |
|  | **Nahuatl_SLP** | 0.00 | 0.06 | 0.05 | 0.00 | 0.01 | 0.00 | 0.02 | 0.00 | 0.00 | 0.00 | **0.10** | 0.00 | 0.02 | 0.00 | 0.01 | 0.00 | 0.00 | 0.00 | 0.00 | 0.01 | 0.07 | 0.00 | 0.00 | - |  |  |  |  |  |  |  | | |  |
|  | **Nahuatl_Edo Mex** | 0.00 | 0.05 | 0.04 | 0.01 | 0.00 | 0.00 | 0.01 | 0.00 | 0.00 | 0.00 | 0.08 | 0.00 | 0.01 | 0.00 | 0.01 | 0.00 | 0.00 | 0.00 | 0.00 | 0.01 | 0.09 | 0.00 | - |  |  |  |  |  |  |  |  | | |  |
|  | **Nahuatl_CDMX** | 0.00 | 0.04 | 0.03 | 0.03 | 0.00 | 0.00 | 0.00 | 0.00 | 0.00 | 0.00 | 0.06 | 0.00 | 0.00 | 0.00 | 0.00 | 0.00 | 0.00 | 0.00 | 0.00 | 0.00 | **0.11** | - |  |  |  |  |  |  |  |  |  | | |  |
|  | **Nahuatl_Mor** | **0.10** | **0.27** | **0.25** | **0.02** | **0.19** | 0.08 | **0.23** | **0.12** | **0.10** | **0.13** | **0.30** | 0.06 | **0.16** | **0.14** | **0.16** | **0.11** | **0.11** | **0.11** | 0.09 | **0.17** | - |  |  |  |  |  |  |  |  |  |  | | |  |
|  | **Nahuatl_Pue** | 0.01 | 0.00 | 0.00 | 0.07 | 0.00 | 0.01 | 0.00 | 0.00 | 0.00 | 0.00 | 0.02 | 0.03 | 0.00 | 0.00 | 0.00 | 0.00 | 0.00 | 0.00 | 0.01 | - |  |  |  |  |  |  |  |  |  |  |  | | |  |
|  | **Otomi** | 0.00 | 0.05 | 0.04 | 0.02 | 0.00 | 0.00 | 0.02 | 0.00 | 0.00 | 0.00 | 0.08 | 0.00 | 0.01 | 0.00 | 0.01 | 0.00 | 0.00 | 0.00 | - |  |  |  |  |  |  |  |  |  |  |  |  | | |  |
|  | **Totonaco** | 0.00 | 0.03 | 0.03 | 0.03 | 0.00 | 0.00 | 0.00 | 0.00 | 0.00 | 0.00 | 0.06 | 0.00 | 0.00 | 0.00 | 0.00 | 0.00 | 0.00 | - |  |  |  |  |  |  |  |  |  |  |  |  |  | | |  |
|  | **Mazahua** | 0.00 | 0.01 | 0.00 | 0.00 | 0.00 | 0.00 | 0.00 | 0.00 | 0.00 | 0.00 | 0.04 | 0.00 | 0.00 | 0.00 | 0.00 | 0.00 | - |  |  |  |  |  |  |  |  |  |  |  |  |  |  | | |  |
|  | **Popoluca** | 0.00 | 0.04 | 0.03 | 0.02 | 0.00 | 0.00 | 0.01 | 0.00 | 0.00 | 0.00 | 0.06 | 0.00 | 0.01 | 0.00 | 0.00 | - |  |  |  |  |  |  |  |  |  |  |  |  |  |  |  | | |  |
| **S** | **Mazateco** | 0.00 | 0.01 | 0.01 | 0.06 | 0.00 | 0.01 | 0.00 | 0.00 | 0.00 | 0.00 | 0.03 | 0.02 | 0.00 | 0.00 | - |  |  |  |  |  |  |  |  |  |  |  |  |  |  |  |  | | |  |
|  | **Chinanteco** | 0.00 | 0.02 | 0.01 | 0.04 | 0.00 | 0.00 | 0.00 | 0.00 | 0.00 | 0.00 | 0.04 | 0.01 | 0.00 | - |  |  |  |  |  |  |  |  |  |  |  |  |  |  |  |  |  | | |  |
|  | **Mixteco** | 0.01 | 0.01 | 0.00 | 0.07 | 0.00 | 0.01 | 0.00 | 0.00 | 0.00 | 0.00 | 0.03 | 0.03 | - |  |  |  |  |  |  |  |  |  |  |  |  |  |  |  |  |  |  | | |  |
|  | **Zapoteco** | 0.00 | 0.09 | 0.07 | 0.00 | 0.02 | 0.00 | 0.04 | 0.01 | 0.00 | 0.01 | **0.12** | - |  |  |  |  |  |  |  |  |  |  |  |  |  |  |  |  |  |  |  | | |  |
|  | **Chontal_Oax** | 0.07 | **0.00** | **0.00** | **0.18** | 0.02 | 0.09 | 0.00 | 0.06 | 0.07 | 0.05 | - |  |  |  |  |  |  |  |  |  |  |  |  |  |  |  |  |  |  |  |  | | |  |
|  | **Mixe** | 0.00 | 0.03 | 0.02 | 0.04 | 0.00 | 0.00 | 0.00 | 0.00 | 0.00 | - |  |  |  |  |  |  |  |  |  |  |  |  |  |  |  |  |  |  |  |  |  | | |  |
|  | **Huave** | 0.00 | 0.04 | 0.03 | 0.00 | 0.00 | 0.00 | 0.00 | 0.00 | - |  |  |  |  |  |  |  |  |  |  |  |  |  |  |  |  |  |  |  |  |  |  | | |  |
| **SE** | **Tojolabal** | 0.00 | 0.03 | 0.02 | 0.03 | 0.00 | 0.00 | 0.00 | - |  |  |  |  |  |  |  |  |  |  |  |  |  |  |  |  |  |  |  |  |  |  |  | | |  |
|  | **Mocho** | 0.01 | 0.00 | 0.00 | 0.09 | 0.00 | 0.02 | - |  |  |  |  |  |  |  |  |  |  |  |  |  |  |  |  |  |  |  |  |  |  |  |  | | |  |
|  | **Kaqchikel** | 0.00 | 0.06 | 0.05 | 0.00 | 0.00 | - |  |  |  |  |  |  |  |  |  |  |  |  |  |  |  |  |  |  |  |  |  |  |  |  |  | | |  |
|  | **Chuj** | 0.00 | 0.00 | 0.00 | 0.06 | - |  |  |  |  |  |  |  |  |  |  |  |  |  |  |  |  |  |  |  |  |  |  |  |  |  |  | | |  |
|  | **Kanjobal** | 0.03 | **0.14** | **0.13** | - |  |  |  |  |  |  |  |  |  |  |  |  |  |  |  |  |  |  |  |  |  |  |  |  |  |  |  | | |  |
|  | **Mam** | 0.03 | 0.00 | - |  |  |  |  |  |  |  |  |  |  |  |  |  |  |  |  |  |  |  |  |  |  |  |  |  |  |  |  | | |  |
|  | **Jakalteko** | 0.04 | - |  |  |  |  |  |  |  |  |  |  |  |  |  |  |  |  |  |  |  |  |  |  |  |  |  |  |  |  |  | | |  |
|  | **Maya** | - |  |  |  |  |  |  |  |  |  |  |  |  |  |  |  |  |  |  |  |  |  |  |  |  |  |  |  |  |  |  | | |  |

Abbreviations: CDMX, Mexico City; CE, Central East; CW, Central West; Edo Mex; Mexico State; Mor, Morelos; N, North; Oax, Oaxaca; Pue, Puebla; S, South; SE, South East; SLP, San Luis Potosi.

Numbers in bold show highest values of *F*_ST_

Square indicates ethnic groups who showed a mayor level of differentiation with a greater number of ethnicities.
